# Nonlinear relationship between glycated hemoglobin and cognitive impairment after acute mild ischemic stroke

**DOI:** 10.1186/s12883-023-03158-x

**Published:** 2023-03-22

**Authors:** Lei Xu, Qin Xiong, Yang Du, Lu-wen Huang, Ming Yu

**Affiliations:** 1 Department of Neurology, Suining Central Hospital, Suining, 629000 China; 2 Department of Internal Medicine, the Third People’s Hospital of Suining, Suining, 629000 China

**Keywords:** HbA1c, Cognitive impairment, Ischemic stroke, Montreal Cognitive Assessment

## Abstract

**Background:**

Stroke is the second most common cause of morbidity and mortality. Even mild stroke survivors have an increased risk of cognitive impairment. Studies have been conducted on the relationship between glycated hemoglobin (HbA1c) and cognitive decline, but the findings have been inconsistent. Therefore, this study examined the link between HbA1c levels and cognitive impairment following acute mild ischemic stroke.

**Methods:**

Data from 311 patients with acute mild ischemic stroke admitted to Suining Central Hospital, Sichuan Province, China, from January 1, 2015, to December 31, 2018, were evaluated. Fasting venous blood was taken to assess HbA1c levels on the day after admission. Cognitive function was assessed using the Chinese version of the Montreal Cognitive Assessment Scale (MoCA) 3–6 months after stroke onset. We used a generalized additive model and smooth curve fitting (penalty spline method) to assess the nonlinear relationship between HbA1c and poststroke cognitive impairment (PSCI).

**Results:**

This study included 311 patients aged 23 to 96 years old (mean age: 67.37 ± 11.92 years), of whom 198 (63.67%) were men. Among the 311 stroke patients, 120 (38.59%) had PSCI. After adjusting for potential confounders, there was a nonlinear relationship between HbA1c and PSCI, with an inflection point of 8.2. To the left of the inflection point, the effect size, 95% confidence interval, and *P* value were 0.87, 0.58 to 1.31, and 0.5095, respectively; however, to the right of the inflection point, these numbers were 1.96, 1.08 to 3.58, and 0.0280.

**Conclusion:**

We found a nonlinear relationship between HbA1c and PSCI. When HbA1c was greater than 8.2%, HbA1c was positively correlated with PSCI.

## Background

Globally, stroke is the second most common cause of morbidity and mortality [1]. Cognitive impairment is a common complication after stroke that has a poor prognosis and places a heavy burden on families and society [2]. Mild stroke with mild clinical symptoms has a good prognosis and no obvious neurological signs. However, research shows an increased risk of developing cognitive impairment even in mild stroke survivors [3]. Therefore, early identification of important risk factors for cognitive impairment in mild stroke will enable clinicians to intervene earlier in high-risk patients.

Glycated hemoglobin (HbA1c) is used as a marker of blood glucose control since it indicates the average level of blood glucose over the previous three months [4]. It is essential for glucose control in patients with diabetes [5] and provides higher test-to-test consistency than individual fasting or postload blood glucose readings [6]. HbA1c and cognitive impairment have been previously studied, but the results have been inconsistent. Several studies have found HbA1c to be a risk factor for cognitive impairment in people with diabetes [7–9]. However, in patients with acute ischemic stroke, the relationship between HbA1c and cognitive impairment has not been studied in depth, and no correlation between the two has been found [10–13]. Therefore, this study examined the link between HbA1c levels and cognitive impairment following acute mild ischemic stroke and identified prospective biomarkers for poststroke cognitive impairment (PSCI) identification and prevention.

## Materials and methods

### Subjects

In this retrospective cohort study, data from 736 patients with acute ischemic stroke admitted to Suining Central Hospital, Sichuan Province, China, from January 1, 2015, to December 31, 2018, were recruited. The following were the criteria for inclusion: 1) patients were 18 years of age and older; 2) patients were hospitalized within 7 days of stroke start; 3) patients had a National Institutes of Health Stroke Scale (NIHSS) score of less than or equal to 3; and 4) patients had acute cerebral infarction confirmed by magnetic resonance imaging (MRI) during hospitalization.

**Fig. 1:**
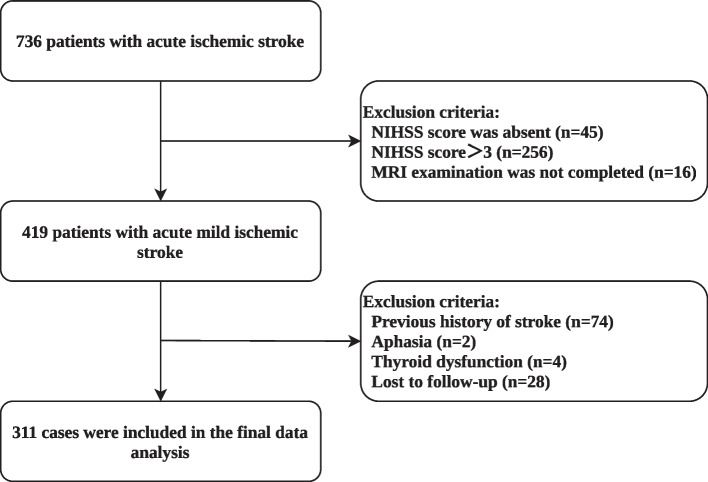
An overview of the selection process

The following were the criteria for exclusion: 1) previous history of stroke; 2) aphasia that made it impossible to assess cognitive function; 3) history of mental problems, neurological diseases, thyroid diseases, autoimmune diseases, or tumors; and 4) prestroke dementia or cognitive impairment. A total of 311 patients with acute mild ischemic stroke were eventually included in the final analysis (as shown in Fig. 1). The ethics committees at Suining Central Hospital approved this study in accordance with the Helsinki Declaration.

### Data collection

#### Covariates

On admission, age, sex, body mass index (BMI), education, smoking status, alcohol use, hypertension, diabetes, atrial fibrillation, and other information were collected. Fasting venous blood was taken on the day after admission to assess fasting plasma glucose (FPG), HbA1c, blood lipid, and uric acid (UA) levels. Within 72 h after hospitalization, MRI was performed. Within 24 h of admission, the NIHSS was used to determine the severity of the stroke, with a score of less than or equal to 3 indicating mild ischemic stroke. At discharge, the modified Rankin scale (mRS) was used to assess functional outcomes.

#### Cognitive assessments

Cognitive function was assessed using the Chinese version of the Montreal Cognitive Assessment Scale (MoCA) 3–6 months after stroke onset [14]. The highest possible MoCA score was 30, and less than 26 was defined as cognitive impairment. A score of less than 25 was classified as cognitive impairment if the number of years of education was less than or equal to 12 years [15, 16].

### Statistical analysis

Data are reported as the mean ± standard deviation (SD) (Gaussian distribution) or median (range) (skewed distribution) for continuous variables and as numbers and percentages for categorical variables. χ2 (categorical variables), one-way ANOVA (normal distribution), or Kruskal–Wallis H test (skewed distribution) were used to detect differences in variables among different HbA1c groups (based on tertiles). To examine the effect of HbA1c on PSCI, we constructed three different models, namely, Model 1 (no covariates were adjusted for), Model 2 (only sociodemographic variables were adjusted for) and Model 3 (covariates are presented in Table 3). A 95% confidence interval was calculated for the effect sizes. We used smooth curve fitting (penalized spline method) to account for nonlinearity between HbA1c and PSCI as well as the generalized additive model (GAM). In addition, a two-piecewise binary logistic regression model was used to further explain the nonlinearity. Next, we performed a subgroup analysis and used the likelihood ratio test to examine subgroup interactions. Modeling was performed with the statistical software packages R (http://www.R-project.org, The R Foundation) and EmpowerStats (http://www.empowerstats.com, X&Y Solutions, Inc., Boston, MA). *P* values less than 0.05 (two-sided) were considered statistically significant.

## Results

### Baseline characteristics of the participants

This study comprised 311 patients aged 23 to 96 years old (mean age: 67.37 ± 11.92 years), including 198 (63.67%) men. The HbA1c ranges for tertiles 1–3 (T1-3) were 4.1–5.4, 5.5–6.3, and 6.4–14.7%, respectively. Significant differences in FPG, homocysteine (HCY), high-sensitivity C-reactive protein (hs-CRP), alcohol consumption, and diabetes mellitus were observed among the groups with different HbA1c levels (Table 1).

**Table 1:** Baseline characteristics of participants

HbA1c tertile, %	Low (4.10–5.40)	Middle (5.50–6.30)	High (6.40–14.70)	*P*-value
No. of subjects	88	119	104	
Age, mean (SD), year	66.12 (13.66)	67.04 (11.67)	68.80 (10.50)	0.281
BMI, mean (SD), kg/m^2^	22.75 (3.15)	23.15 (2.55)	22.99 (2.81)	0.595
HDL, mean (SD), mmol/L	1.45 (0.44)	1.42 (0.35)	1.43 (0.44)	0.837
LDL, mean (SD), mmol/L	2.53 (0.83)	2.61 (0.83)	2.55 (1.24)	0.810
TG, median (min–max), mmol/L	1.55 (0.03–6.22)	1.58 (0.04–7.61)	1.94 (0.03–6.99)	0.349
TC, mean (SD), µumol/L	4.56 (1.54)	4.27 (1.35)	4.45 (1.35)	0.302
FPG, mean (SD), mmol/L	5.32 (1.29)	5.27 (0.99)	8.45 (3.75)	**< 0.001**
HCY, mean (SD), µumol/L	13.53 (6.19)	16.11 (10.10)	13.25 (4.09)	**0.007**
Cr, mean (SD), µumol/L	77.50 (22.90)	77.71 (22.02)	79.33 (41.22)	0.894
BUN, mean (SD), mmol/L	6.08 (2.22)	6.26 (2.35)	6.67 (2.81)	0.240
UA, mean (SD), µumol/L	331.51 (118.72)	334.51 (113.32)	319.11 (99.52)	0.558
hs-CRP, median (min–max), mg/L	3.33 (0.21–704.00)	1.20 (0.15–84.24)	5.80 (0.04–85.69)	** < 0.001**
MOCA, mean (SD)	24.41 (2.17)	24.50 (2.21)	24.21 (2.35)	0.618
Sex, n (%)				0.741
Male	56 (63.64%)	73 (61.34%)	69 (66.35%)	
Female	32 (36.36%)	46 (38.66%)	35 (33.65%)	
Cognitive impairment, n (%)				0.780
No	53 (60.23%)	76 (63.87%)	62 (59.62%)	
Yes	35 (39.77%)	43 (36.13%)	42 (40.38%)	
Education, n (%)				0.843
Undergraduate, college or above	3 (3.41%)	2 (1.68%)	5 (4.81%)	
High school (including technical secondary school)	4 (4.55%)	9 (7.56%)	6 (5.77%)	
Junior high school	17 (19.32%)	24 (20.17%)	23 (22.12%)	
Primary school	36 (40.91%)	51 (42.86%)	46 (44.23%)	
Illiteracy	28 (31.82%)	33 (27.73%)	24 (23.08%)	
Smoking status, n (%)				0.339
Never-smoker	55 (62.50%)	86 (72.27%)	77 (74.04%)	
Past smoker who has quit	14 (15.91%)	16 (13.45%)	15 (14.42%)	
Current smoker	19 (21.59%)	17 (14.29%)	12 (11.54%)	
Alcohol consumption, n (%)				**0.014**
Yes	30 (34.09%)	20 (16.81%)	23 (22.12%)	
No	58 (65.91%)	99 (83.19%)	81 (77.88%)	
Hypertension, n (%)				0.388
Yes	55 (62.50%)	65 (54.62%)	65 (62.50%)	
No	33 (37.50%)	54 (45.38%)	39 (37.50%)	
Diabetes mellitus, n (%)				** < 0.001**
Yes	2 (2.27%)	7 (5.88%)	49 (47.12%)	
No	86 (97.73%)	112 (94.12%)	55 (52.88%)	
Hyperlipidemia, n (%)				0.633
Yes	2 (2.27%)	4 (3.36%)	5 (4.81%)	
No	86 (97.73%)	115 (96.64%)	99 (95.19%)	
Atrial fibrillation, n (%)				0.674
Yes	2 (2.27%)	3 (2.52%)	1 (0.96%)	
No	86 (97.73%)	116 (97.48%)	103 (99.04%)	
mRS score, n (%)				0.080
1	7 (7.95%)	22 (18.49%)	9 (8.65%)	
2	69 (78.41%)	81 (68.07%)	82 (78.85%)	
3	10 (11.36%)	15 (12.61%)	9 (8.65%)	
4	0 (0.00%)	1 (0.84%)	0 (0.00%)	
5	2 (2.27%)	0 (0.00%)	4 (3.85%)	
NIHSS score, n (%)				0.514
0	13 (14.77%)	19 (15.97%)	8 (7.69%)	
1	21 (23.86%)	33 (27.73%)	33 (31.73%)	
2	29 (32.95%)	37 (31.09%)	38 (36.54%)	
3	25 (28.41%)	30 (25.21%)	25 (24.04%)	

*Abbreviations*: *BMI* body mass index, *HDL* high-density lipoprotein, *LDL* low-density lipoprotein, *TG* triglycerides, *TC* total cholesterol, *FPG* fasting plasma glucose, *HbA1c* glycosylated hemoglobin, *HCY* homocysteine, *Cr* creatinine, *BUN* blood urea nitrogen, *UA* uric acid, *hs-CRP* high-sensitivity C-reactive protein, *MOCA* Montreal Cognitive Assessment, *mRS* modified Rankin Scale, *NIHSS* National Institutes of Health Stroke Scale

### Characteristics of the PSCI and non-PSCI groups

Of the 311 recruited stroke patients, 120 (38.59%) of them had PSCI, and 191 had normal cognition. There were significant differences in creatinine (Cr), blood urea nitrogen (BUN), UA, hypertension and atrial fibrillation between the two groups (*P* ≤ 0.05) (Table 2).

**Table 2:** Characteristics of the PSCI and non-PSCI groups

Characteristic	Non-PSCI	PSCI	*P*-value
No. of subjects	191	120	
Age, mean (SD), year	67.13 (11.45)	67.75 (12.66)	0.656
BMI, mean (SD), kg/m^2^	23.08 (2.76)	22.84 (2.89) 22.20	0.455
HDL, mean (SD), mmol/L	1.45 (0.43)	1.41 (0.37)	0.497
LDL, mean (SD), mmol/L	2.58 (1.02)	2.55 (0.92)	0.772
TG, median (min–max), mmol/L	1.80 (0.03–7.61)	1.50 (0.03–6.63)	0.239
TC, mean (SD), µmol/L	4.43 (1.41)	4.39 (1.41)	0.818
FPG, mean (SD), mmol/L	6.19 (2.73)	6.59 (2.86)	0.221
HBAC	6.23 (1.51)	6.56 (2.00)	0.107
HCY, mean (SD), µmol/L	14.47 (5.89)	14.35 (9.64)	0.888
Cr, mean (SD), µmol/L	71.00 (20.41)	82.71 (33.90)	** < 0.001**
BUN, mean (SD), mmol/L	5.93 (2.21)	6.61 (2.61)	**0.019**
UA, mean (SD), µmol/L	253.82 (68.97)	375.44 (105.63)	** < 0.001**
hs-CRP, median (min–max), mg/L	4.00 (0.04–704.00)	3.10 (0.15–84.24)	0.872
MOCA, mean (SD)	23.05 (1.81)	26.49 (0.72)	** < 0.001**
The time from stroke onset to MoCA assessment, mean (SD), month	4.44 (0.99)	4.55 (1.03)	0.346
Sex, n (%)			0.923
Male	122 (63.87%)	76 (63.33%)	
Female	69 (36.13%)	44 (36.67%)	
Education, n (%)			0.424
Undergraduate, college or above	7 (3.66%)	3 (2.50%)	
High school (including technical secondary school)	9 (4.71%)	10 (8.33%)	
Junior high school	35 (18.32%)	29 (24.17%)	
Primary school	86 (45.03%)	47 (39.17%)	
Illiteracy	54 (28.27%)	31 (25.83%)	
Smoking status, n (%)			0.525
Never-smoker	131 (68.59%)	87 (72.50%)	
Past smoker who has quit	27 (14.14%)	18 (15.00%)	
Current smoker	33 (17.28%)	15 (12.50%)	
Alcohol consumption, n (%)			0.551
Yes	47 (24.61%)	26 (21.67%)	
No	144 (75.39%)	94 (78.33%)	
Hypertension, n (%)			**0.047**
Yes	122 (63.87%)	63 (52.50%)	
No	69 (36.13%)	57 (47.50%)	
Diabetes mellitus, n (%)			0.628
Yes	34 (17.80%)	24 (20.00%)	
No	157 (82.20%)	96 (80.00%)	
Hyperlipidemia, n (%)			0.433
Yes	8 (4.19%)	3 (2.50%)	
No	183 (95.81%)	117 (97.50%)	
Atrial fibrillation, n (%)			**0.023**
Yes	1 (0.52%)	5 (4.17%)	
No	190 (99.48%)	115 (95.83%)	
mRS score, n (%)			0.815
1	23 (12.04%)	15 (12.50%)	
2	141 (73.82%)	91 (75.83%)	
3	23 (12.04%)	11 (9.17%)	
4	1 (0.52%)	0 (0.00%)	
5	3 (1.57%)	3 (2.50%)	
NIHSS score, n (%)			0.218
0	25 (13.09%)	15 (12.50%)	
1	53 (27.75%)	34 (28.33%)	
2	57 (29.84%)	47 (39.17%)	
3	56 (29.32%)	24 (20.00%)	

### Relationships between HbA1c and PSCI

To examine the links between HbA1c and PSCI, we utilized a binary logistic regression analysis. Table 3 shows the nonadjusted and adjusted models. In Model 1, HbA1c showed no correlation with PSCI (OR = 0.96, 95% confidence interval (CI): 0.78 to 1.17, *P* = 0.6568). In Model 2 (adjusted for age and sex), the result were not different (OR = 0.94, 95% CI: 0.77 to 1.16, *P* = 0.5811). We also found no connection in Model 3, a fully adjusted model after correcting for other factors (OR = 1.0, 95% CI: 1.00 to Inf, *P* = 1.0000). We also used HbA1c as a categorical variable (tertiles) for sensitivity analysis and found the same pattern (*P* = 1.0000).

**Table 3:** Relationships between HbA1c and PSCI

Exposure	Model 1 OR (95%CI) *P*-value	Model 2 OR (95%CI) *P*-value	Model 3 OR (95%CI) *P*-value
HbA1c	0.96 (0.78, 1.17) 0.6568	0.94 (0.77, 1.16) 0.5811	1.00 (0.00, Inf) 1.0000
HbA1c tertile			
Low	Ref	Ref	Ref
Middle	0.98 (0.44, 2.20) 0.9683	0.97 (0.43, 2.18) 0.9454	1.00 (0.00, Inf) 1.0000
High	0.90 (0.39, 2.10) 0.8157	0.87 (0.37, 2.02) 0.7402	1.00 (0.00, Inf) 1.0000
*P* for trend	0.95 (0.63, 1.44) 0.8126	0.93 (0.61, 1.42) 0.7359	1.00 (0.00, Inf) 1.0000

Model 1: Non-adjusted model

Model 2: Adjusted for Age and Sex

Model 3: Adjusted for Age, Sex, BMI, Education level, Smoking Status, Drinking Status, Hypertension, Diabetes mellitus, Atrial fibrillation, HDL, LDL, TG, TC, FPG, UA, Hyperlipidemia, HCY, Cr, BUN, hs-CRP, mRS, and NIHSS

### Nonlinear connection studies

**Fig. 2:**
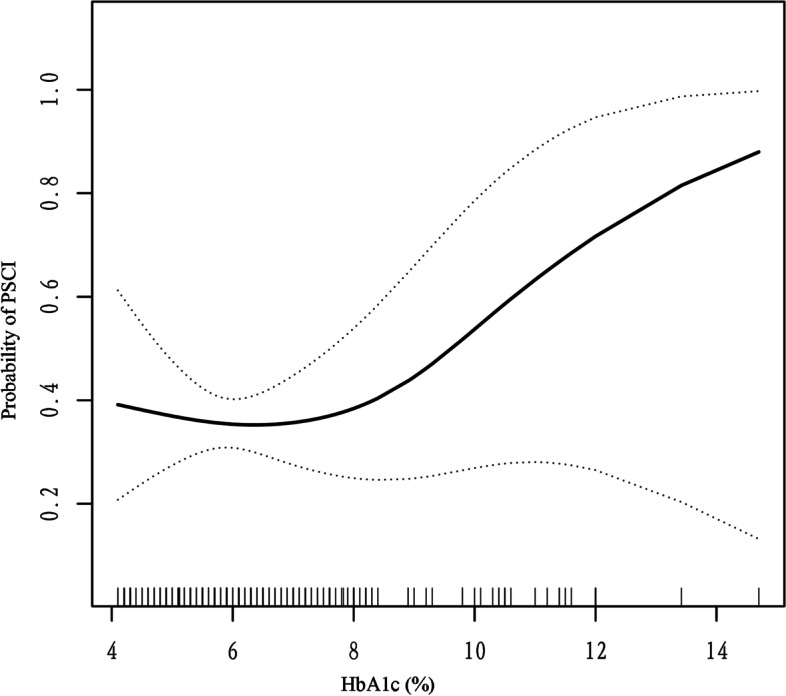
The nonlinear connection studies. The same adjustments were made as in model 3

We investigated the nonlinear relationship between HbA1c and PSCI in this study since HbA1c is a continuous variable (Fig. 2). The relationship between HbA1c and PSCI was discovered to be nonlinear after adjusting for potential confounders. By using a two-piecewise linear regression model, we found the inflection point at 8.2. To the left of the inflection point, the effect size, 95% CI, and P value were 0.87, 0.58 to 1.31, and 0.5095, respectively. However, to the right side of the inflection point, these numbers were 1.96, 1.08 to 3.58, and 0.0280), and we found a positive connection between HbA1c and PSCI (Table 4).

**Table 4:** Threshold effect analysis of HbA1c on PSCI

Outcome	OR, 95%CI, *P*-value
Model 1	
One line slope	1.17 (0.88, 1.55) 0.2722
Model 2	
Infection point	8.2
≤ 8.2	0.87 (0.58, 1.31) 0.5095
> 8.2	1.96 (1.08, 3.58) 0.0280
*P* for log likelihood ratio test	0.044

Adjustments are the same as those in model 3

### The results of the subgroup analysis

By subgroup analysis, we further explored additional risks between HbA1c and PSCI to assess other factors that might influence the results. Age, gender, BMI, education, smoking status, alcohol consumption, hypertension, diabetes mellitus, hyperlipidemia, mRS score and NIHSS score were selected as stratification factors (Table 5). According to the results, the relationship between HbA1c and PSCI was not modified by any of the above influencing factors.

**Table 5:** Relationships between HbA1c and PSCI in various subgroups

Subgroup	No. of subjects	Odds ratio (95% CI)	*P*-value	*P* for interaction
Age, year				0.9746
23—63	103	1.19 (0.80, 1.78)	0.3945	
64—73	100	1.14 (0.75, 1.72)	0.5362	
74—96	108	1.13 (0.77, 1.66)	0.5264	
Gender				0.3913
Male	198	1.25 (0.88, 1.78)	0.2117	
Female	113	1.05 (0.74, 1.49)	0.7701	
BMI, kg/m2				0.6970
15.63—21.61	103	1.04 (0.71, 1.53)	0.8396	
21.64—23.83	100	1.27 (0.85, 1.90)	0.2487	
23.88—33.33	108	1.21 (0.78, 1.89)	0.3905	
Education				0.2517
Undergraduate, college or above	10	0.05 (0.00, 4.26)	0.1910	
High school (including technical secondary school)	19	1.10 (0.30, 4.10)	0.8861	
Junior high school	64	1.03 (0.68, 1.56)	0.8915	
Primary school	133	1.10 (0.88, 1.38)	0.7321	
Illiteracy	85	1.64 (0.99, 2.71)	0.0555	
Smoking status,				0.1512
Never-smoker	218	1.08 (0.93, 1.24)	0.5473	
Past smoker who has quit	45	1.86 (0.95, 3.66)	0.0710	
Current smoker	48	0.78 (0.36, 1.67)	0.5222	
Alcohol consumption				0.6461
Yes	73	1.29 (0.72, 2.33)	0.3974	
No	238	1.13 (0.84, 1.52)	0.4316	
Hypertension				0.4415
Yes	185	1.24 (0.88, 1.75)	0.2250	
No	126	1.06 (0.75, 1.50)	0.7476	
Diabetes mellitus				0.2544
Yes	58	1.34 (0.87, 2.08)	0.1846	
No	253	1.00 (0.71, 1.42)	0.9931	
Hyperlipidemia				0.6520
Yes	11	1.34 (0.60, 2.97)	0.4770	
No	300	1.11 (0.83, 1.48)	0.4737	
mRS score				0.1269
0–2	270	1.17 (0.88, 1.58)	0.2839	
3–4	41	0.65 (0.32, 1.35)	0.2504	
NIHSS score				0.7445
0	40	0.76 (0.28, 2.05)	0.5844	
1	87	1.24 (0.79, 1.96)	0.3477	
2	104	1.20 (0.82, 1.75)	0.3424	
3	80	1.09 (0.74, 1.61)	0.6744	

Above model adjusted for Age, Sex, BMI, Education level, Smoking Status, Drinking Status, Hypertension, Diabetes mellitus, Atrial fibrillation, HDL, LDL, TG, TC, FPG, UA, Hyperlipidemia, HCY, Cr, BUN, hs-CRP, mRS, and NIHSS. In each case, the model is not adjusted for the stratification variable

## Discussion

The connection between HbA1c and PSCI among participants was investigated using generalized linear model (GLM) and GAM models in this study. HbA1c was not linked with PSCI in the fully corrected model, as demonstrated. The same pattern was observed when HbA1c was treated as a categorical variable. However, a nonlinear relationship between HbA1c and PSIC was found, with different correlations on the left and right sides of the inflection point (HbA1c = 8.2%). On the left-hand side of the inflection point, HbA1c showed no significant relationship, but on the right-hand side of the inflection point, HbA1c was positively related to PSIC.

Gong et al. found that 122 (53.5%) of 228 patients with mild stroke who were assessed for cognitive impairment by MoCA 6–12 months after onset (MoCA < 22 was defined as cognitive impairment) developed cognitive impairment [17]. In a Korean study of 301 patients with acute ischemic stroke, 65 patients (21.6%) developed PSCI when cognitive impairment was assessed by the K-VCIHS-NP 3 months after onset [11]. In our study, 38.59% of patients with acute mild ischemic stroke were diagnosed with cognitive impairment 3–6 months after onset, which is different from previous studies. The reason is mainly related to the different evaluation criteria regarding cognitive function.

HbA1c and PSCI have been correlated in previous studies, but the results have been inconsistent. Two previous studies on cognitive impairment in acute ischemic stroke patients did not find a correlation between HbA1c and PSCI [12, 13]. Two other studies of cognitive impairment 3 months after stroke also found no association between HbA1c and cognitive impairment after stroke [10, 11]. However, a cohort study found that HbA1c was an independent risk factor for cognitive impairment 6–12 months after acute mild ischemic stroke by multivariate logistic regression analysis [17]. The present study is the first to identify a curvilinear relationship between HbA1c and cognitive impairment at 3–6 months after mild ischemic stroke. Previous studies that did not find differences in HbA1c between the PSCI and non-PSCI groups did not conduct analyses of nonlinear relationships [10–13].

We employed the GAM to elucidate the nonlinear interactions between HbA1c and PSCI, as well as the generalized linear model to analyze their linear relationship. We found that for every 1% increase in HbA1c greater than 8.2%, there was a 0.96-fold increase in the risk of PSCI. The clinical significance of this discovery is that the link between HbA1c and PSCI can only be seen when HbA1c reaches a particular level. The Mexican Health and Aging Study found that HbA1c ≥ 8% was associated with poorer cognitive performance in older adults with diabetes [18]. Another study found that diabetes was associated with cognitive impairment only when it was poorly controlled (e.g., HbA1c ≥ 7.5%), suggesting that it was the degree of hyperglycemia, rather than diabetes itself, that had a negative impact on cognitive health [19]. The above two studies involved diabetic patients. After adjusting for various confounding factors, our study found that HbA1c > 8.2% was an independent risk factor for PSCI. Our study found that the cut-off point was 8.2, which seems to be much higher than what we expect through basic knowledge. The reason may be related to the small sample size of people with elevated HbA1c.

HbA1c is a commonly assessed parameter that reflects the average blood glucose concentration over the past 8–12 weeks and is a good indicator for evaluating long-term blood glucose control [20]. Elevated HbA1c is caused by increased glycosylation of proteins due to hyperglycemia [21]. Hyperglycemia and dementia have been linked in many studies, both animal and clinical studies, which show that short-term hyperglycemia can lead to learning and memory loss in experimental animals [22–24]. In addition, previous epidemiological studies have reported that hyperglycemia and diabetes status are independently associated with the incidence of dementia [25]. Hyperglycemia is associated with poorer cognitive performance and is caused by dysregulation of insulin and the expression of insulin-degrading enzymes [26, 27]. Intracerebral insulin originates from pancreatic beta cells and relies on efficient IRec-mediated insulin transport across the blood–brain barrier (BBB) to play an important role in cognition, including promoting learning and memory in older adults [28, 29].

This was the first study to identify a curvilinear relationship between HbA1c and cognitive impairment at 3–6 months after mild ischemic stroke. its effect on cognition from our study suggests the effect of average blood glucose may as a vascular risk factor on PSCI, rather than the effect of blood glucose in acute phase on PSCI. Thus, the implication of our study is the influence of pre-stroke blood glucose control on PCSI.

There were certain limitations to our research. First, as this study is a retrospective study, selection bias and lack of data are inevitable. Second, the HbA1c-PSCI relationship cannot be generalized to all ischemic stroke populations due to the exclusion of those with moderate to severe stroke severity. Third, the inclusion of people in western China creates regional and ethnic boundaries. Fourth, those with cardiopulmonary insufficiency were omitted, perhaps underestimating the prevalence of cognitive impairment, and the findings do not apply to this population. Furthermore, neuroimaging factors, including lesion size and location, were not assessed in this investigation.

## Conclusion

In conclusion, we found a nonlinear relationship between HbA1c and cognitive impairment 3–6 months after acute mild ischemic stroke. When HbA1c was greater than 8.2%, HbA1c was positively correlated with PSCI.

## Data Availability

All data generated and analysed during this study for this report are included in this published article and its supplementary information files. Additional study data can be requested from the corresponding author on request.
